# Efficient cyanide sensing using plasmonic Ag/Fe_3_O_4_ nanoparticles†

**DOI:** 10.1039/d3ra06654a

**Published:** 2023-11-09

**Authors:** Razieh Moosavi, Ramin Zibaseresht

**Affiliations:** a Nano Electronic Center of Excellence, Nano Bio Electronic Devices Lab, School of Electrical and Computer Engineering, University of Tehran Tehran Iran; b Biomaterials and Medicinal Chemistry Research Centre, Aja University of Medical Sciences Tehran Iran rzi12@uclive.ac.nz; c Department of Chemistry and Physics, Faculty of Sciences, Maritime University of Imam Khomeini Nowshahr Iran

## Abstract

In the line of our previous studies, we have reported a developed sensitive and selective probe for cyanide detection based on Ag/Fe_3_O_4_ nanoparticles (NPs) with an extremely low limit of detection at the level of ng per milliliter. Herein, we report the improvement of the easy-to-make magnetic silver nanoparticle-based sensor system for cyanide determination in an extended calibration range with higher selectivity and precision. As far as our knowledge is concerned, the detectable linear range from 1.0 nM to 160 μM (0.026 ng mL^−1^ to 4.16 μg mL^−1^) of the improved simple highly precise technique represents the widest assay that has been reported so far. The method is based on strong enhancement of scattered light of the plasmonic nanoparticles and simultaneously cyanide fluorescence quenching. Although the fluorescence of cyanide is highly selective and precise, its intensity is poor. On the other hand, the strongly enhanced Rayleigh signal has a low repeatability. We proposed a method to remove the interference and obtained an effective factor that is directly proportional to cyanide concentration utilizing both above signals simultaneously. In this work, Ag/Fe_3_O_4_ NPs have been synthesized easily using a green preparation method and the NPs were consequently characterized using powder XRD, UV-Vis absorption spectroscopy, transmission electron microscopy (TEM) and energy dispersive X-ray spectroscopy (EDX). A combination of absorption, Rayleigh and fluorescence characteristics were used for detection of cyanide in real samples and an overview of recently reported sensors for cyanide was also provided.

## Abstract 2

In the line of our previous studies, we have reported a developed sensitive and selective probe for cyanide detection based on Ag/Fe_3_O_4_ nanoparticles (NPs) with an extremely low limit of detection at the level of ng per milliliter.

## Graphical abstract

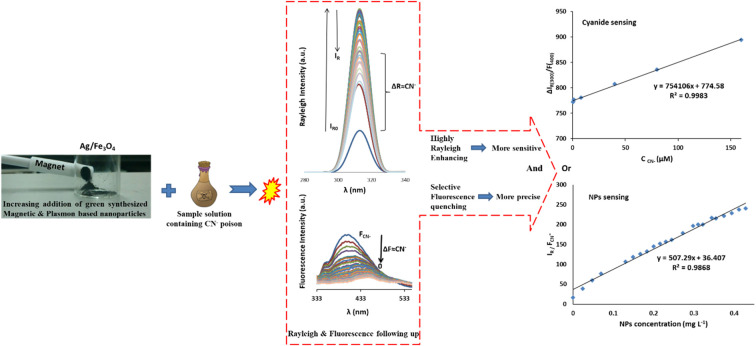

## Introduction

Considerable research into the development of methods for cyanide detection is of prime importance due to the lethal toxicity of cyanide in physiological systems, as well as environmental concerns.^1^ Due to its acute toxicity, cyanide is considered the most dangerous substance for the environment and human life.^2^ Cyanide is still widely used in mining, metallurgy and various chemical industries despite its toxicity, and then inevitably leads to environment pollution. Lethal doses (LD_50_) of cyanide ion (CN^−^) are about 0.5–3.5 mg kg^−1^ of body weight.^3^ Numerous approaches for cyanide sensing have been studied for more than 10 years.^1,2,4–12^ Different methods have been widely used to analyze cyanide such as titrimetric, voltammetric, potentiometric, ion chromatography and universal spectrophotometric techniques.^10,13–15^ A comparison of the performances of the earlier reported works for CN^−^ ion sensing using different detection systems has been reported recently.^16^ Here, Table 1S† presents some of the latest published studies on optical/electrochemical probes for cyanide. The review table covers the most sensitive reports in the field of detecting cyanide ion over the past years (2015–2023). However, some of them show several drawbacks such as narrow pH range, lack of sensing ability in aqueous solution, or not so often could be used in the analysis of environmental samples^17^ despite cyanide's widespread presence in many industrial and municipal wastewaters. In relation to the increased interest in the subject of cyanide poison analysis, researches aim to offer analytical techniques that improves the sensitivity and selectivity with rapidly and simply implementation. Previously, we succeeded to introduce a direct probe for simple detection of trace cyanide concentration in solutions.^15^ The method was based on the large affinity of cyanide in complex formation by Ag NPs and decrement of enhanced Rayleigh signal due to the Ag etching. The use of the strong Ag–CN interaction is an attractive idea for a label free cyanide detection.^15,18–20^ To improve the selectivity and precision of the sensor in an essential unique wide concentration range of cyanide, we, in this report, proposed a new precise dual Rayleigh–fluorescence assay based on a simplest and most basic titrimetric method.

Over the last decade, several types of sensors have been developed on the basis of the plasmonic optical properties of NPs made of noble metals for a wide range of applications in catalysis, optics, measurement of chemical and biological quantities and medical therapeutics.^21–23^ H. Ju group have been reviewed optical sensing of cyanide anions by noble metal nanomaterials.^24^ Metal NPs, especially silver and gold NPs, with the distinct optical and electronic properties have received significant attention for the fabrication of plasmonic sensors.^25–27^ The collective excitations of electrons in the conduction band in resonance with an electromagnetic field of incoming light (coherent oscillation of the metal conduction and free electrons at the surface of metal nanoparticles, in the local enhanced electromagnetic field called surface plasmon resonance, SPR^28^) and consequently an intense excitation signal can enhance weak multi-photon processes leading to surface enhanced spectroscopy that improve the sensitivity dramatically. Surface enhanced spectroscopy, refers to the enhancement of any well-known spectroscopic signals from the molecules at the surface, such as strong plasmon absorption, surface enhanced luminescence, surface enhanced Raman scattering, and surface enhanced Rayleigh scattering.^15^ NP made of noble metals specially in hybrid forms with other nanostructures has been used based on surface enhanced spectroscopies for a variety of applications.^15^ As examples Au/Fe_3_O_4_ dumbbell-shaped heterostructures were used as dual optical/MRI agents during *in vitro* studies of epithelial cells by Xu *et al.*^29^ The Wei group^30^ reported Au/Fe_3_O_4_ usage to make plasmon-resonant nanoparticles and nanostars for magnetomotive imaging. High performance photocatalytic hydrogen production and degradation of levofloxacin by wide spectrum responsive Ag/Fe_3_O_4_ bridged SrTiO_3_/g-C_3_N_4_ plasmonic nano-junctions and Joint effect of Ag and Fe_3_O_4_ were proposed by Kumar in 2018.^31^ Amplification effect of plasmon NPs was suggested widely for detection as sensors and biosensors.^32–35^

Surface enhanced Raman scattering (SERS) is probably one of the most powerful related techniques currently available for chemical and biological sensing analysis, biomedical applications, food and environmental safety control and also for molecular structural detection of specific molecules.^27,36–38^ The most SERS active metal is silver, while SERS enhancement factors as high as 10^14^–10^15^ have been described for silver NPs,^39,40^ resulted in detection limits as low as a single molecule.^39,41^ Also, the immense sensitivity achieved in SERS permits the ultrasensitive and selective detection of biomolecules^38,42^ as well as concomitants.^37,43^

In recent years Rayleigh scattering as a highly sensitive, simple and quick analytical technique has also shown its broad applications.^15^ Strong plasmonic enhancement of light scattering by gold or silver nanoparticles, made nanotechnology capable to detect and sense molecular information even in single living cells^44,45^ that improve medical diagnosis. Beside the plenty useful usage of Rayleigh scattering, there have been studies on particle size measurement as well.^45,46^ Rayleigh and Raman (a weaker analogue) scattering are closely similar processes in which light is scattered by atoms or molecules. Mentionable that Rayleigh scattering is stronger and more sensitive than Raman vibrations which are usually weak. The efficiency of Rayleigh scattering for silver NPs is about 10^6^ times higher than the fluorescence efficiency of a fluorescent dye molecule with the distinct advantage that silver NPs neither blink nor bleach against fluorescent probes.^47^

At the same time, the integration of magnetic nanoparticles (MNPs) with analytical methods has opened a new path for sensing, purification, and quantitative analysis.^48–50^ MNPs compared with noble metal and semiconductor NPs, have neither no fluorescence nor surface plasmon absorption.^51^ But, due to their high refractive indices and molecular weights despite their small sizes, make them a powerful enhancer for plasmonic responses.^52^ Therefore, the use of MNPs as SPR amplification factor was the subject of many researches.^52,53^ Also, it is particularly beneficial to design hybrid NPs that combine the magnetic properties together with plasmonic activity^54^ which allow simultaneous monitoring of analytes and magnetic control of the NPs (to avoid from their probably further pollution).^19,37,55,56^ The research of metal nano-based fluorescent sensors is a particularly active research field due to their intrinsic advantages of the fluorescent methods.^19,30,57^ According to Zhai *et al.*,^19^ bifunctional Au–Fe_3_O_4_ NPs for sensitive and selective turn-on fluorescent detection of cyanide based on the inner filter effect field provided a sensitive strategy. Song, *et al.* in 2020,^30^ gathered magneto-optical properties of Ag modified Fe_3_O_4_ NPs with all-in-one sensing platforms.

In the line of our interests in the development of methods for sensing toxic ions,^15^ we have proposed an improved hybrid Rayleigh–fluorescence method for cyanide monitoring using dual-functional magnetic-optical nanosensor. The prepared silver magnetite-based nanocomposite (Ag/Fe_3_O_4_) showed a remarkable optical property, enabling a significant improvement in the sensitivity due to the surface plasmon enhancing of both Ag and magnetic NPs. The sensor also has the unique property of strong rapid interaction of nanocomposite with cyanide ions, providing resulting to fabricate a low cost label-free sensor for fast, selective and highly precise detection of ultra-trace amounts of cyanide. The proposed method showed the widest linear concentration range for cyanide detection compared to those reported previously (Table 1S†). As a competition study, the obtained results showed not only the performance of our proposed sensor in ultra-trace cyanide determinations, but also it presents a direct simple approach for cyanide monitoring in a wide detection range over four orders of magnitudes that require not such an expensive instrumentation or special skill, which is crucial and beneficial to meet the requirements in the environmental monitoring.^17^ The new assay is more applicable among the studies (Table 1S†) as improved optical probe for cyanide sensing with favorable sensitivity and selectivity, high working pH span and repeatability, and high detection range ability for practical precise operation.

## Experiment

### Reagents and instrumentation

All chemicals were of analytical reagent grade and used as supplied without further purifications. All solutions were freshly prepared with double distilled water. Rayleigh and fluorescence spectra and their intensities at a given wavelength were obtained using a PerkinElmer luminescence spectrometer (LS50B). Transmission electron microscopy was performed on a JEOL, JEM-2100 electron microscope. The chemical compositions were examined using energy-dispersive spectroscopy (EDS). X-ray diffraction (XRD) studies were carried out on a Philips X'Pert-MPD X-ray diffractometer at the ambient temperature. A Metrohm 713 pH-meter and a 40 kHz RoHS universal ultrasonic cleaner bath 35 W were used for pH adjustments and cleaning, respectively. Fourier transform infrared (FTIR) spectra of the samples were obtained using a Nicolet, IR 100 FT-IR spectrometer in the KBr matrix.

### Preparation of the Ag/Fe_3_O_4_ nanoparticles

Magnetic Fe_3_O_4_ NPs were synthesized through alkaline precipitation of ferric and ferrous iron salts from their acidic aqueous solutions as before.^58,59^ The Ag/Fe_3_O_4_ nanoparticles were prepared, using glucose as a mild green reducing agent and AgNO_3_ in water, similar to our previously work.^15^

### Cyanide sensing using the Ag/Fe_3_O_4_ nanocomposite

Solutions with varying concentration of Ag/Fe_3_O_4_ nanoparticles (0 to 0.5 mg L^−1^) were added to cyanide solution and spectroscopically analyzed using the main bands at 313, 400 and 625 nm (excitation wavelength of 300 nm) which can be assigned to strong resonance Rayleigh scattering, cyanide fluorescence, and second-order Rayleigh scattering (2Rayleigh scattering) bands, respectively. The enhancement in Rayleigh scattering signal (Δ*I*_R_) is proportional to the nanoparticles and cyanide concentration in a wide range. For greater impact of present assay, the applicability of the new rapid, selective and highly precise method in real samples has been investigated. CAUTION: care should be taken during contact with any solutions containing cyanide.^20^ Prior studies demonstrated that Ag NPs and iron oxide both in the hybrid nanostructure, amplify the Rayleigh signals and enhance the sensitivity of the cyanide probe whereas separately usage, which emphasize the role of iron oxides besides Ag NPs enhancement effect in addition to the reusability capability of the nanocomposite (ESI Fig. S4 and S5† from ref. 15).

## Results and discussion

### Characterization of the Ag/Fe_3_O_4_ nanoparticles

**Fig. 1:**
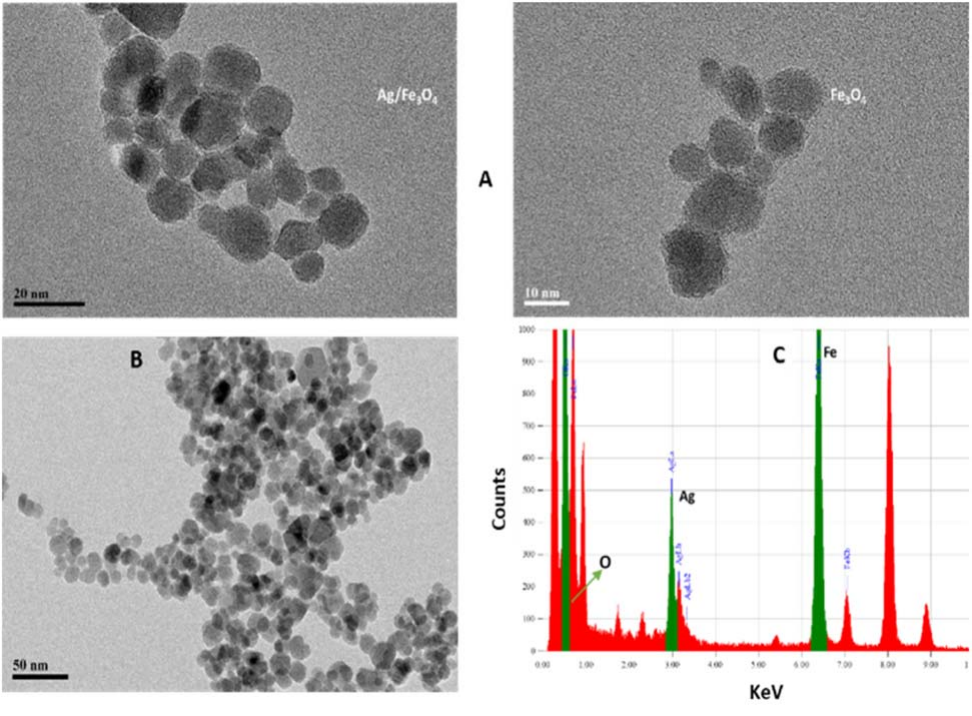
(A) TEM images (B) XRD patterns from the Ag/Fe_3_O_4_ nanoparticles (red) and Fe_3_O_4_ (black) and (C) EDX analysis of the nanocomposite.

**Fig. 2:**
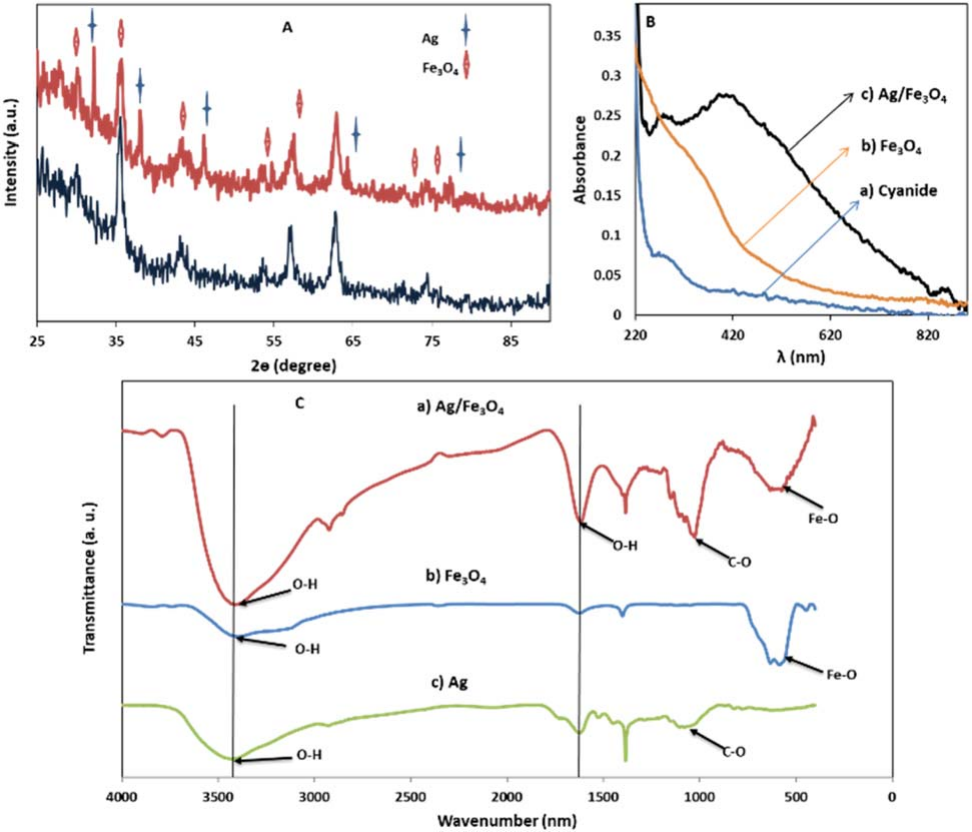
(A) XRD patterns for Ag and Fe_3_O_4_ samples; (B) UV-vis absorption spectra of (a) cyanide, (b) Fe_3_O_4_ nanoparticles, and (c) Ag/Fe_3_O_4_ nanocomposite, 1 mg L^−1^; (C) FTIR spectra for (a) Ag/Fe_3_O_4_, (b) Fe_3_O_4_ and (c) Ag samples.

Transmission electron microscopy (TEM) shows an average particle size of below 20 nm for the Fe_3_O_4_ particles (Fig. 1A). Using the Scherrer equation^58^ and powder XRD spectrum (Fig. 2A) a crystallite size of 15 nm was calculated. Consistent with our previous works,^15,60^ as sensing using nanostructures are highly dependent upon the size of the particles, by addition of optimized amounts of Ag to Fe_3_O_4_ nanoparticles in a mild condition, Ag NPs grown randomly beside Fe_3_O_4_ NPs and partially covered the particles in an efficient size. The narrowing and decreasing of the Fe_3_O_4_ peaks (Fig. 2A) are ascribed to the nanoparticles' smaller crystalline sizes during Ag addition.^15^ TEM images with different magnifications (Fig. 1) and EDX (energy dispersive X-ray spectroscopy) (Fig. 1C) of the related selected TEM image (Fig. 1B) shows the coverage and confirmed the presence of Ag beside iron oxide NPs in the prepared nanocomposites. Further support come from the XRD (Fig. 2A) and also appearance of the Ag typical surface plasmon resonance band in the absorption spectra of the nanocomposite after adding silver to Fe_3_O_4_ NPs (Fig. 2B). In the XRD pattern (Fig. 2A), curves clearly show two sets of strong diffraction peaks, indicating that the as-synthesized products are composite materials with good crystallinity and high purity. And also there was no shift in the positions of the reflection peak of particles compare to the bare Fe_3_O_4_. In Fig. 2A the diffraction peaks at 2*θ* = 38.1°, 44.3°, 64.4° and 77° is related to the (111), (200), (220) and (311) planes of the face-centered cubic lattice of silver.^61^ The diffraction peaks at 30°, 35.5°, 43.1°, 53.4°, 57° and 62.8° should be related to Fe_3_O_4_, corresponds to the cubic inverse spinel structure (ICDD No. 88-0315). The optical properties of repeatedly prepared magnetic nanoparticles were examined and the results almost were the same. Therefore, based on our observations, the reproducibility for the preparation of the silver magnetite nanoparticles was satisfactory here and in our previous studies.^15^

FTIR spectra (Fig. 2C) of Fe_3_O_4_ NPs exhibited vibrations in the region 400–700 cm^−1^, which can be attributed to the vibrations of Fe–O (A peak corresponds to the stretching vibration associated with an oxygen–metal absorption band appears here (Fig. 2C)).^60,61^ The band at about 1600 cm^−1^ in Fig. 2C can be associated to O–H stretching and bending vibrations typical of the water molecule.^61^

Besides, the peaks around 1050 cm^−1^ can be belong to the C–O bond stretching mode. Also for Ag NPs, the FTIR indicated the presence of peak nearby 1700, which may correspond to stretching vibrations of glucose, as reductant. Comparing the FTIR spectra of Fe_3_O_4_ and Ag NPs with the Ag/Fe_3_O_4_, the presence of Fe_3_O_4_ is clearly indicated by presence of peaks in the 400–700 cm^−1^ region. The peaks corresponding to O–H and C–O stretching vibration are also present in the nanocomposite indicating the presence Ag NPs and its strong interaction with Fe_3_O_4_ as seen in Fig. 2C.^60^

### The cyanide Rayleigh–fluorescence sensing assay

**Fig. 3:**
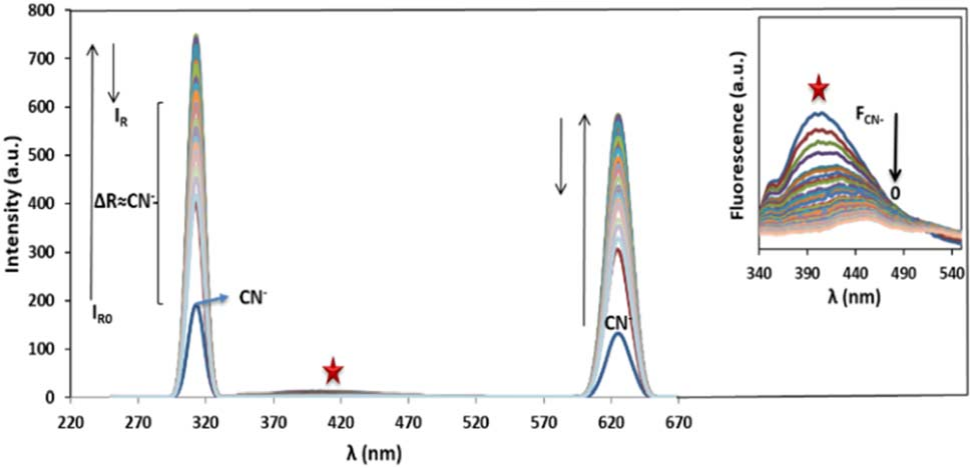
Rayleigh scattering and fluorescence upon addition of Ag/Fe_3_O_4_ nanoparticles, [CN^−^] = 4.16 μg mL^−1^, pH = 8.0, *λ*_ex_ = 300 nm (inset: the zoom fluorescence spectra of red star position).

Ag/Fe_3_O_4_ NPs with different amounts (ranging from 0 to 0.5 mg L^−1^) were mixed gradually with exact concentrations of cyanide solutions (known concentration of CN^−^ in different calibration range experiments from 1 nM to 160 μM). Resonance Rayleigh scattering, cyanide fluorescence as well as second-order Rayleigh scattering (2Rayleigh scattering) bands in the visible range of the solutions were then analyzed immediately for cyanide sensing (Fig. 3). The fluorescence intensity of cyanide was decreased upon addition of the NPs to the cyanide solution, while, at the same time, the Rayleigh scattering and 2Rayleigh scattering were sharply increased reaching a maximum signal before decreasing again with higher NP loads. It is noteworthy that the difference between the wavelengths related to the fluorescence and Rayleigh scattering was large enough (Δ*λ* = 87 nm for Rayleigh and Δ*λ* = 225 nm for 2Rayleigh), which contributes to the accurate measurement of the intensities of the two peaks.

Based on our observations these signals were proportional to the cyanide concentrations; therefore, we concluded that, in order to improve the sensing assay, both fluorescence and Rayleigh scattering signals for cyanide determination should be used. The cyanide fluorescence signals were not appearing to be so strong, and in spite of high selectivity, its sensitivity (as well as the linear range of related calibration curve) was limited.

Also in the presence of interferences or matrix effects, despite the fluorescence of special concentration of cyanide remains constant, the Rayleigh scattering was slightly increased and its reproducibility and precision was relatively poor. Considering these two factors is a key for effective cyanide sensing. That means by addition of Ag/Fe_3_O_4_ NPs to cyanide solution, the fluorescence emission of cyanide decreased and at the same time the Rayleigh scattering was proportionally increased.

Ag could be etched by cyanide selectively, liberated from the NPs; thus, rapid complexation and decreasing in fluorescence is occurred. Fluorescence signal quenching brings a good selectivity and reproducibility for cyanide detection. Meanwhile, increasing the enhanced Rayleigh intensity related to the cyanide fluorescence decreasing, resulted in high sensitivity. By addition of the NPs to some extent to cyanide solution titrimetrically, the fluorescence intensity of cyanide disappears. The first zero point was achieved by monitoring the instrumental fluorescence signal at *λ*_em_ = 400 nm, while the Δ*F* remains constant with tolerance *t* ≤ ±5%. Under such condition, the enhancement in Rayleigh scattering signal was linearly proportional to the cyanide concentration. In overall, besides highly enhancing in Rayleigh scattering signal because of the presence of small amount of Ag and iron oxides NPs in the probe, selective fluorescence quenching could serve as the basis for direct, sensitive and highly selective cyanide determination in a wide concentration range. In particular, iron oxide parts could be recovered due to the reproducible properties under an external magnetic force which in turn helps to remove their probably pollution effects. Also, the iron oxide parts could be simply re-used in the preparation of nanostructures with Ag. We should note that the iron oxides were highly magnetic, even after they formed a nanostructure with silver.^52^ As the reversibility parameters could have established to term the composite as a good probe, the recyclable magnetic nanoparticles particles were employed again by extraction with a magnet and washing, to create silver iron oxide nanostructures in order to use in a separate experiment for cyanide detection. The results showed approximately the same efficiencies (For determination of cyanide solution containing 3 mg L^−1^ ion subsequently by recovered composite 2.85 mg per L CN^−^ was detected with RSD below 5% for two repeated tries, and more than 95 percent recovery was achieved) (note: the magnetic particles loss is little due to their high strong magnetic property and collected simply for future use). Also the re-used NPs were gathered for another cycle repeatability after the first sensing experiment, by separation and washing the composite without any silver modification. The results showed that the efficiency decreases dramatically.

This can be attributed as a result from etching of Ag by cyanide ion that liberate the silver NPs from the nanostructure to form a stable complex by cyanide and therefore decreasing the Rayleigh scattering.

### The proposed mechanism

**Fig. 4:**
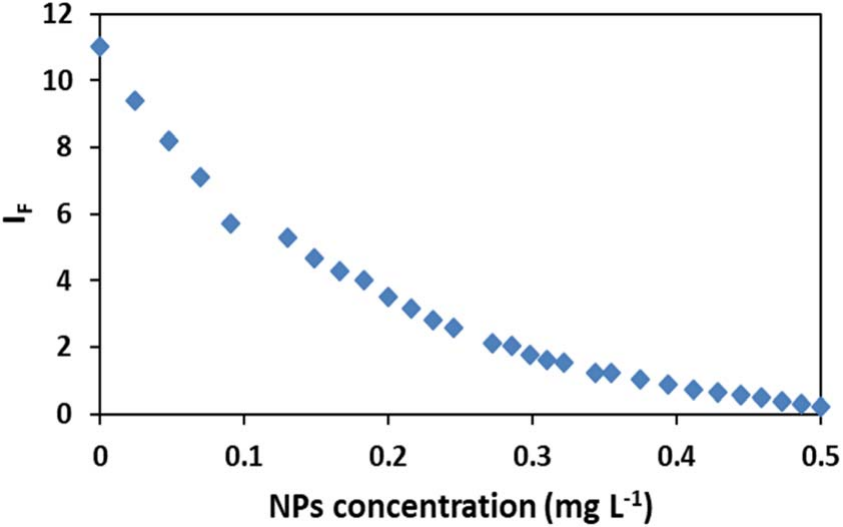
Effect of nanoparticles concentration on the fluorescence intensity *I*_F_, [CN^−^] = 4.16 μg mL^−1^, pH = 8.0.

The fluorescence intensity (*I*_F_) was determined using the data obtained from the spectra (Fig. 3) and it showed that the *I*_F_ value was directly proportional to the concentration of NPs (Fig. 4). The decrease of fluorescence intensity indicates etching of Ag/Fe_3_O_4_ NPs through either the dissolution of silver by cyanide ions^15^ or their adsorption. It also shows some form of quenching process occurring when cyanide ions are placed at a relatively short distance from a metal particle possessing a strong plasmon field and the electrons of the cyanide are also participated in the excitation/emission interact with the field.

The level of the fluorescence decreasing depends upon the field strength and the excitation and emission wavelengths of the fluorophore.^62–65^ Another probable mechanism that we proposed might be the adsorption of cyanide on iron oxides and iron hydroxides, which is in consistent with the mechanism of cyanide toxicity due to the CN^−^ strong binding to the active Fe atom in cytochrome *c* oxidase and inactivating oxidative respiration^66^ which shows the tendency of cyanide to Fe.

In addition, results have also shown a relationship between the enhanced Rayleigh intensity (*I*_R_) and the NPs concentration. *I*_R_ was increased in some orders of magnitude relative to the decrease of fluorescence signal, resulted in improving sensitivity of the method. Considering the formula relation *I*_R_ = *KI*_0_*cM*,^67^ the scattering intensity (*I*_R_) will be proportional to the concentration of the solution (*c*), when the incident light intensity (*I*_0_), the molecular weight of the particle (*M*) and *K* are constant.

Furthermore, comparing the light scattering spectra of cyanide itself with that of silver nanoparticle cyanide conjugates (in Fig. 2), it could be because of the assembly of NPs, the interparticle plasmon coupling and formation cyanide complexes in the solution which play crucial roles in the generation of the Rayleigh changes.^68^

In this study, by addition of nanoparticles to the cyanide solution until the fluorescence quenching become complete, a good linear relationship between the Rayleigh enhancement {Δ*I*_R_ = *I*_R(313 nm)_ − *I*_R0(313 nm)_} and the cyanide concentration was obtained in the range of 1.0 nM to 160 μM (calibration equation *y* = 7 541 06*x* + 774.58, *R*^2^ = 0.9984). The reproducibility of the present method was evaluated. The relative standard deviation (RSD) for six replicate measurements of a typically solution containing 80 μM CN^−^ was 1.2%, illustrated that the response toward cyanide was highly precise. In order to appraising the potential application of our proposed method, quantitative determination of cyanide in tap and waste water real samples was investigated. Credible recoveries (Table 1), suggested the accuracy and reliability of the present method in practical applications.

**Table 1:** Recovery data for five repeated cyanide detection in spiked water samples

Water sample	CN^−^ added (μM)	CN^−^ detected^a^ (μM)	Recovery (%)	RSD (%), *n* = 5
Tap water	0	—	—	—
	80	79 ± 1.5	98.8	1.9
Waste water	0	—	—	—
	80	78 ± 1.1	97.8	1.4

a Mean ± standard deviation (*n* = 5).

**Fig. 5:**
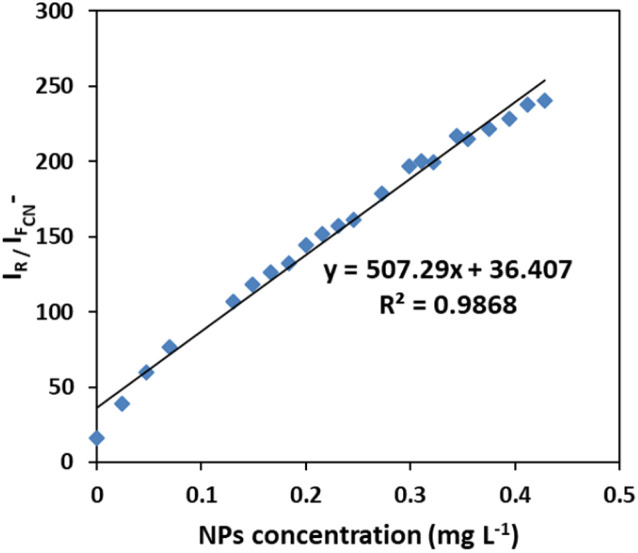
Calibration curve for NPs sensing. Linear plot of ratiometric Rayleigh at 313 nm *versus* fluorescence at 400 nm to the concentration of Ag/Fe_3_O_4_ nanoparticle.

It is notable that taking the ratiometric spectra intensities of scattering and fluorescence of plasmonic Ag/Fe_3_O_4_ nanoparticles in this assay, had a good linear relationship with the nanocomposite concentration from 0.02 to 0.5 mg L^−1^ (Fig. 5), therefore it could be also proposed a reliable detecting method for NPs by which exhibited a good selectivity and a low detection limit of 6 ng mL^−1^.

### The effect of pH

**Fig. 6:**
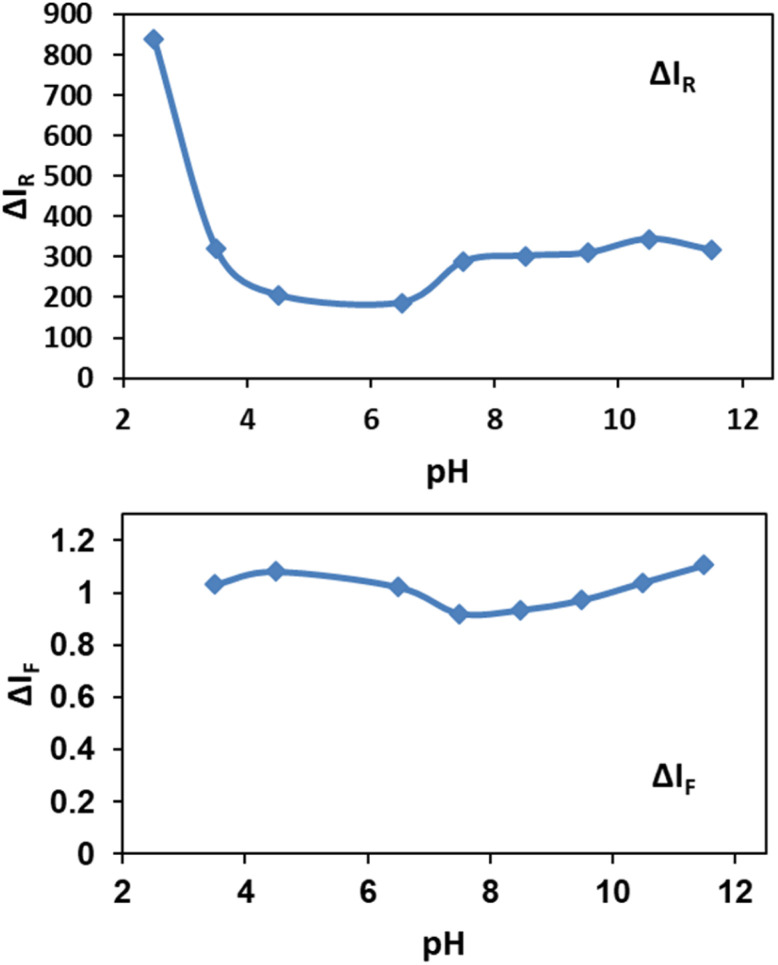
Effect of pH on Δ*I*_R_ and Δ*I*_F_, by addition of 0.03 mg per L nanoparticles.

In order to obtain favorable working pH range, the effect of pH in the range of 2.5 to 11.5 on spectral intensities was investigated (Fig. 6).

As shown in Fig. 6, pH below 3.0 for solutions were not suitable for determination assay because NPs were soluble in highly acidic media. Also cyanide ion as a weak acid could be protonated at pH below 3.0 by abstracting the available protons and forms hydrocyanic acid (HCN) which has a lower tendency for silver etching.^69^ For Rayleigh scattering less pH-sensitive methods are recommended. Here, the obtained results in Fig. 6 show the relatively constant pH values in the range between 7.0 to 11.5. Also by addition of NPs to the cyanide solutions, decreasing in fluorescence of cyanide in the range of 3.5 to 11.5 is nearly constant. Although Jaszczak *et al.* concluded that most cyanide analysis sampling protocols specify the preservation of samples at a pH of 12 or higher,^1^ the results indicate the robustness of our sensing system in a wide pH range. Based on our observations, a pH of 7.5 was selected for the further experiments.

### The effect of wavelength-dependency

As equation below shows that the intensity of Rayleigh scattering is proportional to the sixth power of the particle size of a molecule and *λ*_ex_^−4^.*σ*_e_ = (2π^5^/3)(*d*^6^/*λ*^4^)(*n*^2^ − 1/*n*^2^ + 2)^2^where *λ* is the wavelength of the light, *n* is the refractive index of the particle, and *d* is the diameter of the particle. The enhanced scattering observed for the NPs can be attributed to resonance of the scattering wavelength with the surface plasmon resonance.^70^ For the purpose of obtaining the best Rayleigh spectra signal, different excitation wavelengths in the range of 250 nm to 700 nm were examined. The extent of fluorescence decrease was also excitation wavelength-dependent.

In order to obtain the maximum Rayleigh increasing while the most fluorescence decreasing, the optimum *λ*_ex_ value as 300 nm was selected in our studies.

### Response time

**Fig. 7:**
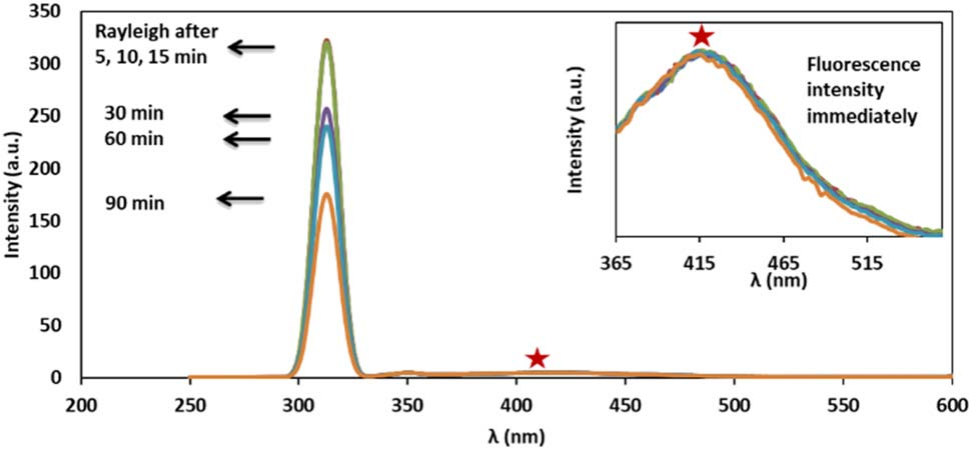
Time dependency investigation of Rayleigh scattering and fluorescence of solution containing 3 mg L^−1^ of cyanide after addition of 0.08 mg L^−1^ of Ag/Fe_3_O_4_ nanoparticles, immediately up to 195 min, pH 8.0; inset the closer fluorescence spectra.

The dependency of time was investigated by monitoring the spectra in the presence of 3 mg L^−1^ of cyanide solution and 0.08 mg L^−1^ of Ag/Fe_3_O_4_ nanoparticles (Fig. 7). We observed that fluorescence peaks were time independent and remained unchanged after hours (Fig. 7).

**Fig. 8:**
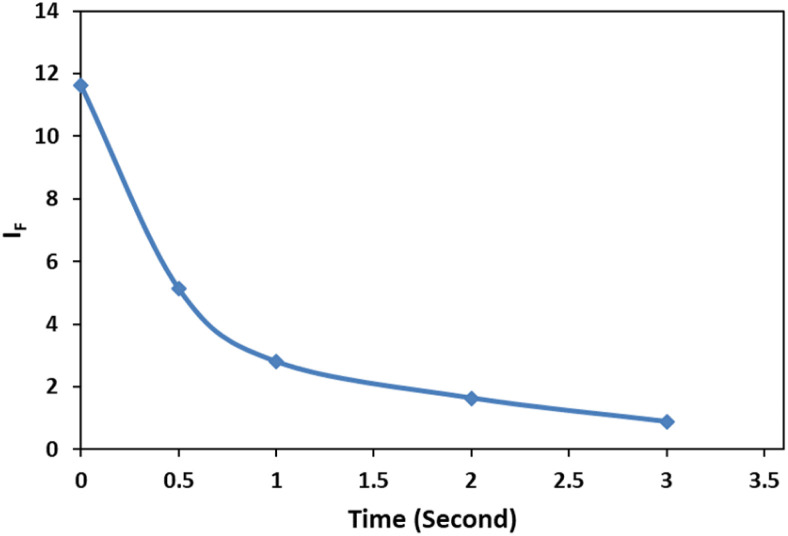
Fluorescence intensity (*I*_F_) monitoring, [CN^−^] = 4.16 μg mL^−1^, pH 8.0; immediately up to 3 seconds.

However, after 15 min, Rayleigh scattering is decreased gently. To identify the results, the Rayleigh scattering and the fluorescence spectra were repeated at least 3 times for more than one batch of nanoparticles and the relative standard deviation of the changes was below ±1%. Also the result revealed that the fast interaction between the composite and cyanide ion can be completed immediately after the addition of NPs (one of the method advantages is a quick response time that mean decreasing in fluorescence and increasing in Rayleigh scattering were occurred simultaneously in seconds (Fig. 8)). Fig. 8 shows the fluorescence intensity (IF) monitoring of cyanide, immediately up to 3 seconds related to Fig. 4 conditions (it seems that the interaction complete mostly in first seconds and follows first order linear regression model (*y* = −0.8039*x* + 2.1433, *R*^2^ = 0.943) with slope about −0.8).

Therefore, spectral data could be recorded immediately upon the addition of NPs, indicated that the method proved a rapid capability for cyanide determination. Furthermore, since the fluorescence quenching was time independent (Fig. 7), this study gave more precise and repeatable results than our previous work.^15^

### Interfering study for other ions

**Fig. 9:**
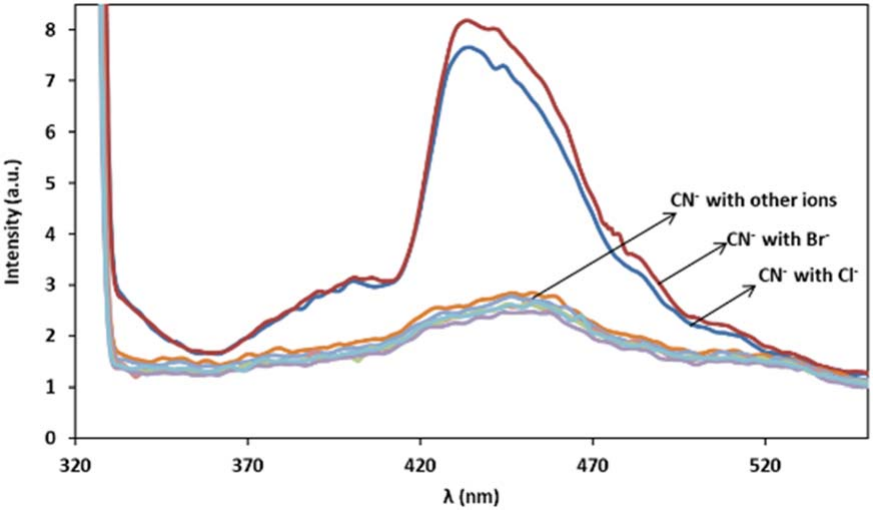
Fluorescence monitoring of cyanide in the presence of more than 1000-fold excess of various species after 10 min, *λ*_ex_ = 300 nm, pH 8.0.

In order to assess the ions selectivity, a variety of relevant ions were screened, and fluorescence spectral changes upon addition of the ions including SCN^−^, NO_3_^−^, SO_3_^2−^, H_2_PO_4_^−^, CrO_4_^−^, OH^−^, Cl^−^, Br^−^, S^2−^, S_2_O_3_^2−^, Zn^2+^, Na^+^ and K^+^ to the mixtures of 3 mg L^−1^ of cyanide solution and 0.08 mg L^−1^ of Ag/Fe_3_O_4_ solution at room temperature were studied (Fig. 9).

The results confirmed that the most species that we investigated did not interfere even when present 1000-fold excess than CN^−^, and induce no significant changes in fluorescence emission intensity of cyanide. In the case of halides there is a fluorescence change observation only in the presence of high interferences concentrations (*e.g.* by more than about 10^3^-fold excess of the bromide and 10^6^ of chloride *via* cyanide) and the reason may be due to the lower solubility product constant, *K*_sp_ for silver halide preparation (in about 25 °C *K*_sp_ for AgCN is 2.2 × 10^−16^, AgCl: 1.82 × 10^−10^ and AgBr: 5.0 × 10^−13^). That means that using higher halides concentration, Ag^+^–halide formation could compete with Ag^+^–CN^−^ interaction. Therefore, it proved that the method of cyanide sensing that we employed, provided an excellent selectivity over the environmentally common interface ions because of the highly selective Ag^+^–CN^−^ complexation formation and so on (the formation constant, *K*_f_(β 2), for [Ag(CN)_2_]^−^ at 25 °C is about 1.0 × 10^21^). As scrutiny relative to our previous work, higher selectivity over competitive interferences and much better precision due to the reproducible selective fluorescent signal was achieved.

## Conclusions

In this research work, we have reported an easy “green” preparation of plasmonic–magnetic of Ag/Fe_3_O_4_ NPs as an approved probe for analysis and recognition of different levels of cyanide contamination in a wide toxic concentration range of 1.0 nM to 160 μM. The new probe displays remarkable dual changes in fluorescence and highly analytical signal of surface-enhanced Rayleigh scattering by nanoparticles addition. Δ*I*_R_ increment proportional to the fluorescence decreasing was used, as a beneficial improvement in the precision, accuracy and selectivity of cyanide sensing.

We also reported an improvement of the easy-to-make magnetic silver nanoparticle-based sensor system for cyanide determination in an extended calibration range with higher selectivity and precision. We proposed a method to remove the interference and obtained an effective factor that is directly proportional to cyanide concentration utilizing both above signals simultaneously.

We also demonstrated that the sensor exhibited more effective applicability in real sample that can be used for detecting lethal cyanide in practical water systems. The new strategy led to other advantages of minimal pH dependence, fast response time, low detection limit (as low as 1 nM)/high sensitivity, high precision and selectivity in presence of other competitive environmental ions and also low cost preparation. Therefore, improved performance (as LOD, linear concentration range and selectivity) is achieved in this improved study. In summary, in this work, Ag/Fe_3_O_4_ NPs have been synthesized easily by green preparation method and the NPs were consequently characterized using powder XRD, UV-Vis absorption spectroscopy, transmission electron microscopy (TEM) and energy dispersive X-ray spectroscopy (EDX). Combinations of absorption, Rayleigh and fluorescence characteristics were used for the detection of cyanide in real samples.

## Author contributions

Razieh Moosavi: conceptualization; methodology; formal analysis; investigation; writing – review & editing and Ramin Zibaseresht: project administration; resources; supervision; writing – review & editing.

## Conflicts of interest

There are no conflicts to declare.

## Supplementary Material

RA-013-D3RA06654A-s001
