# Supplementary material for: Efficient cyanide sensing using plasmonic Ag/Fe3O4 nanoparticles
Source: RSC Adv. 2023 Nov 9;13(47):33120–8. doi: 10.1039/d3ra06654a (PMC10633889; doi:10.1039/d3ra06654a)
Supplement: RA-013-D3RA06654A-s001 [file RA-013-D3RA06654A-s001.pdf]

Electronic Supplementary Material (ESI) for RSC Advances.
This journal is © The Royal Society of Chemistry 2023

Supplementary material

Efficient cyanide sensing using plasmonic Ag/Fe_3O_4 nanoparticles

Razieh Moosavi[1,2], Ramin Zibaseresht[2,3*]

[1]*Nano Electronic Center of Excellence, Nano Bio Electronic Devices Lab, School of Electrical and Computer Engineering, University of Tehran, Tehran, Iran.*

[2]*Biomaterials and Medicinal Chemistry Research Centre, Aja University of Medical Sciences, Tehran, Iran*

[3]*Department of Chemistry and Physics, Faculty of sciences, Maritime University of Imam Khomeini, Nowshahr, Iran.*

Table 1S. Performance summary of most sensitive scientific reported cyanide sensors with significant analytical features in the literature

Method	Minimum reported LOD[b] (nmol L^{-1})	LDR[a] (µmol L^{-1})	Publish year	Ref.
Cysteamine-decorated gold nanoparticles for plasmon-based colorimetric on-site sensors using the smart-phone color ratio and for catalytic reduction of 4-nitrophenol	159	-	2023	1
Smartphone-Based Attomolar Cyanide Ion Sensing Using Au-Graphene Oxide Cryosoret Nanoassembly and Benzoxazolium-Based Fluorophore in a Surface Plasmon-Coupled Enhanced Fluorescence Interface	1	-	2023	2
Ratiometric orange fluorescent and colorimetric highly sensitive imidazolium-bearing naphthoquinolinedione-based probe	1 and 0.13 pM		2023	3
Synthesis of novel 3-hydroxy-2-naphthoic hydrazones as selective chemosensors for cyanide ions	8.2	-	2023	4
A benzoxazole-based fluorescent 'off-on-off' probe for cascade recognition of cyanide and Fe^{3+} ions	23		2023	5
Turn-on fluorescent and colorimetric sensor for cyanide and acetate-based Schiff base compound of 2,2′-((1E,11E)-5,8-dioxa-2,11-diazadodeca-1,11-diene-1,12-diyl)bis(4-((E)-phenyldiazenyl)phenol)	2.1		2023	6
Colorimetric, fluorometric, and electrochemical sensing of cyanide ion in aqueous media using merocyanine-ferrocene conjugate	UV: 2.28, fluorescence spectroscopy: 1.48, and CV: 0.61	-	2023	7
Fluorometric and colorimetric sensor	1.2	0–10 µL	2023	8

by pyridinium scaffolds: live cell imaging and real sample analysis	Not exactly mentioned			
Silicon nanoparticles as a fluorometric probe for sensitive detection of cyanide ion and its application in C. elegans bio-imaging	0.90	0 - 33	2023	9
Electron-deficient moiety regulated structure: an efficient strategy for the design of a highly sensitive cyanide "turn-on" fluorescent probe	58	0.025–40.0	2023	10
Bis-BODIPY-based fluoride and cyanide sensor mediated by unconventional deprotonation of C−H proton	83	-	2022	11
Fluorene based fluorescent and colorimetric chemosensors	0.07 & 0.15	0–0.1	2022	12
Anoptical sensor in aqueous solution and living cells with a fluorogenic naphthalene based receptor (NFS)	210	-	2022	13
Spiropyran and spironaphthoxazine based opto-chemical probes for instant ion detection	SPOH (91) and SNO (94)	-	2022	14
A robust gold nanocluster-peroxyoxalate chemiluminescence system	0.021	0.096–4.8 nM	2022	15
Dual anion colorimetric and fluorometric sensing of arsenite and cyanide ions involving MLCT and CHEF pathways	0.37	0-100	2022	16
Fluorescent lysozyme gold-aryl bioconjugates	-	1 to 12	2022	17
Aggregation induced emission based fluorenes as dual-channel fluorescent probes for smartphones and logic gates applications	0.50, 0.51 & 0.57	0-0.5	2022	18
Development of AIEE active fluorescent and colorimetric probe for the solid, solution, and vapor phase detection	6.17	-	2022	19
Sequential colorimetric sensor via the complexation−decomplexation mechanism based on sugar pyrazolidine-3,5-dione	480	-	2022	20
Selective flurogenic chemosensor for cyanide	234 & 11	-	2021	21
Coumarin-based chemosensor	57.9	-	2021	22
Fluorescence sensing based on Au-modified upconversion nanoassemblies	153	0 to 200	2021	23
Complexation–Decomplexation mechanism with Di(bissulfonamido)spirobifluorene for copper(II) and cyanide sensing	390	0 to 10	2021	24
Mercuric cation-graphene quantum	310	5.0–15.0	2021	25

dots for fluorescence switching detections of cyanide and ferricyanide				
Functional dye based optical sensors by Naphthalene Flanked Diketopyrrolopyrrole	500 & 50	0.05 to1 & 0.5 to 10	2021	[26]
Origami paper analytical assay based on metal complex sensor for fire survivors	400	1-100	2021	[27]
Recognition in a wide pH range by a trifluoroacetamido based metal–organic framework	280	-	2021	[28]
Tetra-imidazole functionalized pyrene for constructing Co-MOF and cyanide sensing	89.3	0-27	2021	[29]
Rational design of an ICT-based chemodosimeter with aggregation-induced emission for colorimetric and ratiometric fluorescent detection	17	0 to 20	2021	[30]
An imine based probe for fluorescent cyanide sensing with red-emission in solid and solution phases	500	0 to 25	2021	[31]
A sensitive and selective BINOL based ratiometric fluorescence sensor	189	0-20	2021	[32]
Gold nanoparticles-decorated paper-based sensor for rapid cyanide detection in water	7680	-	2021	[33]
A flexible salamo-based colorimetric and proportional chemical sensor	128	0-20	2021	[34]
"OFF-ON" fluorescence bisphenol–based cyanide sensing	380	0–60	2021	[35]
Controllable and reversible sensing cyanide ion using dual-functional Cu (II)-based ensemble	In order of nM	0–2500 0–1800	2021	[36]
Aggregation-induced emission-based material	67		2021	[37]
A chemosensor derived from benzamide hydrazones	2150-5837	2.15 to 17.5	2020	[38]
Cyanide chemosensors based on 3-dicyanovinylpyrazolo [1, 5-a] pyrimidines: effects of peripheral 4-anisyl group substitution on the photophysical properties	300 in DPPa to 170 in DPPc	-	2020	[39]
Visual and colorimetric detection based on the morphological transformation of gold nanobipyramids into gold NPs	1.58	1 -15	2020	[40]
Rationally designed imidazole derivative as colorimetric and fluorometric sensor	5300 & 41	-	2020	[41]
Multi-Stimuli Responsive FRET Processes of BiFluorophoric AIEgens in an Amphiphilic Copolymer	260	0-80	2020	[42]
A coumarin-connected carboxylic indolinium sensor for cyanide detection	4.44×10^5	-	2020	[43]

Rapid 'color to concentration' cyanide quantification using paper-based sensing chip	860	0−20	2020	44
Electrochemical Sensor Based on Silver Nanoparticles/Multi-walled Carbon Nanotubes Modified Glassy Carbon Electrode	4	0.1 to 210	2020	45
Ratiometric sensing using dual-emissive Au nanoclusters	10	0.02-1	2020	46
Phenothiazine-based visual and fluorescent sensor	11	-	2020	47
turn-off fluorescent detection using dicyanovinyl-substituted phenanthridine fluorophore	39.3	2-5	2020	26
Sensing of aluminum and cyanide ions utilizing a bis-phenol a based fluorogenic probe	4.81	-	2020	48
Spontaneous optical response by a reactive binding site probe	9.28 & 0.61	-	2020	49
ON–OFF" optical sensor based on hydrogen bonding and water assisted aggregation induced emission	8.2	Up to 0.21	2020	50
Triphenylamine-thiophene dyad as a Chemodosimeter	42.4	1–100	2020	51
Stepwise assembly of turn-on fluorescence sensors in multicomponent metal–organic frameworks	50	0 to 2.67	2020	52
On-site detection by colorimetric and fluorogenic sensors: Smartphone and test strip applications	450	5 to 35	2020	53
Aryl Ethynylpyrene as fluorescent sensor	66 & 87	-	2020	13
turn-off fluorescent detection using dicyanovinyl-substituted phenanthridine fluorophore	39.3	2 to 5	2020	26
Fluorescence "turn-ON" nanosensor using supramolecular hybrid of graphene quantum dots and cobalt pyrene-derivatized phthalocyanine	0.5	0.01-0.05	2019	54
Sensor based on anion–π interaction-driven electron transfer	870	0 to 20	2019	55
Furan/thiophene-based fluorescent hydrazones as fluoride and cyanide	1075	-	2019	56
A dual-mode sensor for colorimetric and turn-on fluorescent detection	226	0-100	2019	57
A carbazole-based colorimetric and fluorescent sensor with aggregation induced emission	67.4	0-10	2019	41
A colorimetric and ratiometric fluorescent probe	339	2-12	2019	58
A colorimetric and fluorometric oligothiophene-indenedione-based sensor	31.3	0-10	2019	59
A turn-on fluorescent chemosensor	350	-	2019	60

based on aggregation-induced emission				
Stimuli-responsive gold nanoparticles modified with poly (N,N-dimethylaminoethyl methacrylate)	0.0047	10^{-5}-10^{-3} & 0.04–320	2019	[61]
Dicyanovinyl-substituted D-A type dithienylethenes: Synthesis, photochromism and colorimetric sensing	250	-	2019	[62]
Fluorescence turn-on chemodosimetric sensing by cyanovinylterpyridine modified phthalonitrile and subphthalocyanine	94	-	2019	[63]
A mitochondria-targeted ratiometric fluorescent probe	0.17	0 to 18	2019	[64]
Colorimetric and fluorometric chemosensors for cyanide detection via ICT off in aqueous solution	3600 & 4200<br>500 & 600	-	2019	[65]
An easy to make chemoreceptor for ratiometric fluorescent detection in food	0.96 mM	Up to 160 mM	2019	[66]
Colorimetric sensor with an organic probe	195	-	2019	[67]
A coumarin chalcone compound as red fluorescent probe	0.31	-	2018	[68]
nucleophilic addition and hydrogen-bond interaction	3 & 9.8	Up to 0.01 & 0.033	2018	[69]
Paper-based headspace extraction combined with digital image analysis	26.92	0.11-3.85	2018	[70]
Copper NPs as a fluorescent probe	370	0.5-18	2017	[71]
Nitrogen and sulfur co-doped graphene quantum dots for an inner filter effect-based cyanide sensor	520	10-500	2017	[72]
Electrochemical sensor by gold NPs decorated carbon ceramic electrode	90	0.5-14	2017	[73]
Fluorescence sensor which in turn inhibit 2-way ESIPT	600	-	2017	[74]
Photoluminescent copper nanoclusters	5	-	2017	[75]
Glassy carbon electrode modified with graphene oxide and titanium dioxide probe	100	0.1-60	2017	[76]
Cyanide Mapping via Surface Plasmon Spectroscopy of Single Au–Ag Nanoboxes	1	Up to 40 nM	2017	[77]
Bimetallic gold-silver nanoclusters sensor	138	0.005-10<br>& 20-50	2017	[78]
Nano biosensor by Ag@Au core–shell nanoparticle (NP)/iridium(III) complex-based sensing platform	36	0.05-80	2017	[79]
IR 786 perchlorate (IR-786) as an optical sensor in organic media and on solid surfaces	500	0.5-8	-2016-	[80]
on-off ratiometric fluorescence probe based on perturbation of the interaction between gold nanoclusters	75	0.1–220	2016	[81]

and a copper (II)-phthalocyanine complex				
Water-soluble fluorescent probe	17	0.2–15	2016	82
Gold nanodots probe	150	0.29 -8.87	2016	83
Spectrophotometric by Improved ninhydrin-based reagent	308	0.78- 15.38	2016	84
An NIR molecule-based "On–Off" fluorescent and colorimetric Sensor	0.46	6-20	2016	85
Nano-sized imprinted polymer synthesis for carbon nanotube impregnated carbon paste electrode sensor	8000	10–100000	2016	86
Chemiluminescence sensor by gold NPs	13.6	0.017 – 1.33	2016	87
Gold NP-based dual fluorescence–colorimetric sensor	100	0-50	2016	88
Rapid turn-on fluorescence sensor	19.6	-	2016	89
Displacement of the glutathione ligand of (GSCbl) by cyanide	1000	1–20	2016	90
Dicanovinyl-based colorimetric and fluorescent sensor	14	Up to 5	2016	91
Colorimetric method by Ag@Au core–shell NPs	160	0.4-32	2016	92
Hexagonal cadmium oxide nanodisks as efficient scaffold	1400	10–80	2016	93
Amperometric inhibition biosensors	30	0.1–58.6	2015	94
Conductometric sensor using imidazole based receptor	10	0.01-1	2015	95
Probe based on Ag/Fe_3O_4 NPs	1.54	0.005-0.05& 0.05-2.31	2015	96
Improved Ag/Fe_3O_4 NP sensor	~0.4	0.001 to 160		This work

[a] Linear dynamic range, [b] Limit of detection

References

1. R. Rajamanikandan, K. Shanmugaraj, M. Ilanchelian and H. Ju, *Chemosphere*, 2023, **316**, 137836.
2. V. S. K. Cheerala, K. M. Ganesh, S. Bhaskar, S. S. Ramamurthy and S. C. Neelakantan, *Langmuir*, 2023.
3. A. Kumar, W. Hur, G. H. Seong and P. S. Chae, *Analytica Chimica Acta*, 2023, **1267**, 341376.
4. R. D. Alharthy, I. Urooj, M. Tasleem, M. Khalid, M. A. Asghar, S. I. Khan, M. Ajmal, N. Ahmed and Z. Shafiq, *RSC advances*, 2023, **13**, 15208-15221.
5. N. Tohora, M. Mahato, T. Sultana, S. Ahamed, S. Ghanta and S. K. Das, *Journal of Photochemistry and Photobiology A: Chemistry*, 2023, **442**, 114807.
6. M. Saremi, A. Kakanejadifard, M. Ghasemian and M. Adeli, *Spectrochimica Acta Part A: Molecular and Biomolecular Spectroscopy*, 2023, **292**, 122397.
7. I. Kathuria, P. Sharma and S. Kumar, *Journal of Photochemistry and Photobiology A: Chemistry*, 2023, 115081.
8. K. Jamuna, S. Gayathri, S. Sivakumar and B. Ashokkumar, *Sensors & Diagnostics*, 2023, **2**, 337-346.
9. J. K. Alagarasan, S. Shasikala, S. Ganesan, M. Arunachalam, U. Manojkumar, S. Palaninaicker, D. D. Nguyen, S. W. Chang, M. Lee and H.-M. Lo, *Environmental Research*, 2023, **224**, 115402.

10. H. Wang, Z. Liu, T. Xie, L. Wang, Q. Xu, M. Chen, Z. Zhang, Z. Ma, S. Fan and H. Wu, *Sensors and Actuators B: Chemical*, 2023, **379**, 133218.
11. W. Saiyasombat, U. Eiamprasert, T. Chantarojsiri, K. Chainok and S. Kiatisevi, *Dyes and Pigments*, 2022, **206**, 110643.
12. M. T. Waseem, H. M. Junaid, S. Majeed, A. M. Khan, T. Mahmood and S. A. Shahzad, *Microchemical Journal*, 2022, **173**, 107018.
13. B. Yilmaz, M. Keskinates, Z. Aydin and M. Bayrakci, *Journal of Photochemistry and Photobiology A: Chemistry*, 2022, **424**, 113651.
14. S. Sanjabi, J. K. Rad and A. R. Mahdavian, *Journal of Photochemistry and Photobiology A: Chemistry*, 2022, **424**, 113626.
15. L. Li, T. Yang, J. Yang and X. Zhang, *Sensors and Actuators B: Chemical*, 2022, **353**, 131038.
16. S. A. Kumar, *Journal of Molecular Structure*, 2022, **1250**, 131677.
17. J. B. Parambath, M. Hameed and A. A. Mohamed, *Chemical Papers*, 2022, **76**, 3619-3626.
18. S. Majeed, M. T. Waseem, H. M. Junaid, G. S. Khan, S. Nawazish, T. Mahmood, A. M. Khan and S. A. Shahzad, *RSC advances*, 2022, **12**, 18897-18910.
19. S. Majeed, M. T. Waseem, G. S. Khan, H. M. Junaid, M. Imran, S. Nawazish, T. A. Khan, T. Mahmood and S. A. Shahzad, *Analyst*, 2022, **147**, 3885-3893.
20. J. Isaad and A. El Achari, *Journal of Molecular Structure*, 2022, **1252**, 132151.
21. H. Muniyasamy, C. Chinnadurai, M. Nelson, A. M. Kubendran, K. Sukumaran, A. Balasubramaniem, M. Sepperumal, S. Ayyanar, M. Govindasamy and A. Ghfar, *Journal of Molecular Liquids*, 2021, **334**, 116076.
22. T. Devendhiran, K. Kumarasamy, M.-C. Lin and Y. X. Yang, *Inorganic Chemistry Communications*, 2021, **134**, 108951.
23. C. Sun and M. Gradzielski, *Analyst*, 2021, **146**, 2152-2159.
24. K. Silpcharu, S. Soonthonhut, M. Sukwattanasinitt and P. Rashatasakhon, *ACS omega*, 2021, **6**, 16696-16703.
25. N. Kongsanan, N. Pimsin, C. Keawprom, P. Sricharoen, Y. Areerob, P. Nuengmatcha, W.-C. Oh, S. Chanthai and N. Limchoowong, *ACS omega*, 2021, **6**, 14379-14393.
26. S. Manickam and S. K. Iyer, *RSC advances*, 2020, **10**, 11791-11799.
27. A. Sheini, M. D. Aseman and M. M. Bordbar, *Scientific Reports*, 2021, **11**, 1-11.
28. A. Rana, C. Gogoi, S. Ghosh, S. Nandi, S. Kumar, U. Manna and S. Biswas, *New Journal of Chemistry*, 2021, **45**, 20193-20200.
29. Y. Liu, Q. Qiu, X. Zhang, K. Huang and D. Qin, *Journal of Solid State Chemistry*, 2021, **300**, 122258.
30. S. D. Padghan, L.-C. Wang, W.-C. Lin, J.-W. Hu, W.-C. Liu and K.-Y. Chen, *ACS omega*, 2021, **6**, 5287-5296.
31. C. Nandhini, P. S. Kumar, K. Poongodi, R. Shanmugapriya and K. P. Elango, *Journal of Molecular Liquids*, 2021, **327**, 114833.
32. S. Munusamy, S. Swaminathan, D. Jothi, V. P. Muralidharan and S. K. Iyer, *RSC advances*, 2021, **11**, 15656-15662.
33. M. L. Budlayan, J. P. Lagare-Oracion, L. D. Rosa, M. J. Rodriguez, J. Manigo, A. Alguno, E. Austria, S. Arco, J. Patricio and C. Deocaris, *Advances in Natural Sciences: Nanoscience and Nanotechnology*, 2021, **12**, 025007.
34. G.-H. Liu, Z.-Z. Chen, Y.-H. Deng and W.-K. Dong, *Journal of Photochemistry and Photobiology A: Chemistry*, 2021, **414**, 113271.
35. Z. Ullah, P. M. Sonawane, T. S. Nguyen, M. Garai, D. G. Churchill and C. T. Yavuz, *Spectrochimica Acta Part A: Molecular and Biomolecular Spectroscopy*, 2021, **259**, 119881.
36. J. Wang, J. He, J. Zhang, Z. Chen, J. Liang and L. Chen, *Spectrochimica Acta Part A: Molecular and Biomolecular Spectroscopy*, 2021, **252**, 119526.

37. G. A. Zalmi, D. N. Nadimetla, P. Kotharkar, A. L. Puyad, M. Kowshik and S. V. Bhosale, *ACS omega*, 2021, **6**, 16704-16713.
38. S. N. Kodlady, B. Narayana, B. Sarojini, S. N. Karanth and B. Gauthama, *Supramolecular Chemistry*, 2020, **32**, 433-444.
39. A. Tigreros, J.-C. Castillo and J. Portilla, *Talanta*, 2020, **215**, 120905.
40. T. Sasikumar and M. Ilanchelian, *New Journal of Chemistry*, 2020, **44**, 4713-4718.
41. Q. Zou, F. Tao, H. Wu, W. Y. William, T. Li and Y. Cui, *Dyes and Pigments*, 2019, **164**, 165-173.
42. P. Q. Nhien, W.-L. Chou, T. T. K. Cuc, T. M. Khang, C.-H. Wu, N. Thirumalaivasan, B. T. B. Hue, J. I. Wu, S.-P. Wu and H.-C. Lin, *ACS applied materials & interfaces*, 2020, **12**, 10959-10972.
43. S. Cheng, X. Pan, M. Shi, T. Su, C. Zhang, W. Zhao and W. Dong, *Spectrochimica Acta Part A: Molecular and Biomolecular Spectroscopy*, 2020, **228**, 117710.
44. H. Singh, G. Singh, D. K. Mahajan, N. Kaur and N. Singh, *Sensors and Actuators B: Chemical*, 2020, **322**, 128622.
45. H. Zhang, D. Sun and T. Cao, *Int. J. Electrochem. Sci*, 2020, **15**, 3434-3444.
46. H. Yang, Y. Yang, S. Liu, X. Zhan, H. Zhou, X. Li and Z. Yuan, *Analytical and Bioanalytical Chemistry*, 2020, **412**, 5819-5826.
47. F. A. Al-Zahrani, R. M. El-Shishtawy, A. M. Asiri, A. M. Al-Soliemy, K. A. Mellah, N. S. Ahmed and A. Jedidi, *BMC chemistry*, 2020, **14**, 1-11.
48. D. Aydin, *Microchemical Journal*, 2020, **159**, 105477.
49. J. H. Park, R. Manivannan, P. Jayasudha and Y.-A. Son, *Spectrochimica Acta Part A: Molecular and Biomolecular Spectroscopy*, 2020, **233**, 118190.
50. R. Nazarian, H. R. Darabi, K. Aghapoor, R. Firouzi and H. Sayahi, *Chemical Communications*, 2020, **56**, 8992-8995.
51. Y. Zhao, L. Feng, X. Meng and J. Guan, *Dyes and Pigments*, 2020, **183**, 108713.
52. J. Li, S. Yuan, J. S. Qin, J. Pang, P. Zhang, Y. Zhang, Y. Huang, H. F. Drake, W. R. Liu and H. C. Zhou, *Angewandte Chemie*, 2020, **132**, 9405-9409.
53. S. Erdemir and S. Malkondu, *Talanta*, 2020, **207**, 120278.
54. O. J. Achadu and T. Nyokong, *Dyes and Pigments*, 2019, **160**, 328-335.
55. G. Men, W. Han, C. Chen, C. Liang and S. Jiang, *Analyst*, 2019, **144**, 2226-2230.
56. N. Saini, C. Wannasiri, S. Chanmungkalakul, N. Prigyai, V. Ervithayasuporn and S. Kiatkamjornwong, *Journal of Photochemistry and Photobiology A: Chemistry*, 2019, **385**, 112038.
57. Z. Guo, Q. Niu, Q. Yang, T. Li and H. Chi, *Analytica Chimica Acta*, 2019, **1065**, 113-123.
58. L. Hou, F. Li, J. Guo, X. Zhang, X. Kong, X. T. Cui, C. Dong, Y. Wang and S. Shuang, *Journal of Materials Chemistry B*, 2019, **7**, 4620-4629.
59. Z. Guo, T. Hu, T. Sun, T. Li, H. Chi and Q. Niu, *Dyes and Pigments*, 2019, **163**, 667-674.
60. K. Deng, L. Wang, Q. Xia, R. Liu and J. Qu, *Sensors and Actuators B: Chemical*, 2019, **296**, 126645.
61. Z. Alinejad, M. Raeesi and A. R. Mahdavian, *Talanta*, 2019, **204**, 198-205.
62. Z. Li, Y. Song, Y. Dai, Y. Pei, Z. Lu and H. Guo, *Optical Materials*, 2019, **95**, 109235.
63. J. B. Arockiam and J. S. Park, *Spectrochimica Acta Part A: Molecular and Biomolecular Spectroscopy*, 2019, **207**, 112-117.
64. Y. Zhu, K. Wang, W. Song, B. Dong, S. Zhao, R. Guan, Z. Li, Y. Sun, D. Cao and W. Lin, *Sensors and Actuators B: Chemical*, 2019, **294**, 283-290.
65. P. G. Rao, B. Saritha and T. S. Rao, *Journal of Photochemistry and Photobiology A: Chemistry*, 2019, **372**, 177-185.
66. P. S. Kumar, P. R. Lakshmi and K. P. Elango, *New Journal of Chemistry*, 2019, **43**, 675-680.
67. R. Bhaskar and S. Sarveswari, *Inorganic Chemistry Communications*, 2019, **102**, 83-89.
68. Y. Sun, Y. Shan, N. Sun, Z. Li, X. Wu, R. Guan, D. Cao, S. Zhao and X. Zhao, *Spectrochimica Acta Part A: Molecular and Biomolecular Spectroscopy*, 2018, **205**, 514-519.

69. K. Wang, X. Yu, Y. Zhu, M. Xing, M. Liang, Y. Sun, D. Cao, R. Guan and Z. Liu, *Journal of Molecular Structure*, 2018, **1173**, 647-652.
70. M. Saraji and N. Bagheri, *Sensors and Actuators B: Chemical*, 2018, **270**, 28-34.
71. S. Momeni, R. Ahmadi, A. Safavi and I. Nabipour, *Talanta*, 2017, **175**, 514-521.
72. C. Chen, D. Zhao, T. Hu, J. Sun and X. Yang, *Sensors and Actuators B: Chemical*, 2017, **241**, 779-788.
73. M. Shamsipur, Z. Karimi and M. A. Tabrizi, *Microchemical Journal*, 2017, **133**, 485-489.
74. S. B. Roy, A. Maity and K. K. Rajak, *Inorganic Chemistry Communications*, 2017, **76**, 81-86.
75. J. Cang, C.-W. Wang, P.-C. Chen, Y.-J. Lin, Y.-C. Li and H.-T. Chang, *Analytical Methods*, 2017, **9**, 5254-5259.
76. R. Hallaj and N. Haghighi, *Microchimica Acta*, 2017, **184**, 3581-3590.
77. P. Wang, Y. Bai, C. Yao, X. Li, L. Zhou, W. Wang, A. M. El-Toni, J. Zi, D. Zhao and L. Shi, *Analytical chemistry*, 2017, **89**, 2583-2591.
78. L. Tian, Y. Li, T. Ren, Y. Tong, B. Yang and Y. Li, *Talanta*, 2017, **170**, 530-539.
79. Z.-Z. Dong, C. Yang, K. Vellaisamy, G. Li, C.-H. Leung and D.-L. Ma, *ACS sensors*, 2017, **2**, 1517-1522.
80. B. Barare, I. Babahan, Y. M. Hijji, E. Bonyi, S. Tadesse and K. Aslan, *Sensors*, 2016, **16**, 271.
81. Z. Shojaeifard, B. Hemmateenejad and M. Shamsipur, *ACS Applied Materials & Interfaces*, 2016, **8**, 15177-15186.
82. M. La, Y. Hao, Z. Wang, G.-C. Han and L. Qu, *Journal of Analytical Methods in Chemistry*, 2016, **2016**.
83. N. Vasimalai and M. T. Fernandez-Arguelles, *Nanotechnology*, 2016, **27**, 475505.
84. A. Surleva, M. Zaharia, O. Pintilie, I. Sandu, L. Tudorachi and R. V. Gradinaru, *Environmental Forensics*, 2016, **17**, 48-58.
85. S. Zhao, H. Liu, F. Wu and L. Zhu, *Chemistry Letters*, 2016, **45**, 570-572.
86. T. Alizadeh, R. E. Sabzi and H. Alizadeh, *Talanta*, 2016, **147**, 90-97.
87. N. Bagheri, N. Djafarzadeh and J. Hassanzadeh, *Analytical Sciences*, 2016, **32**, 317-322.
88. C. Cheng, H.-Y. Chen, C.-S. Wu, J. S. Meena, T. Simon and F.-H. Ko, *Sensors and Actuators B: Chemical*, 2016, **227**, 283-290.
89. T. B. Wei, G. T. Yan, H. Li, Y. R. Zhu, B. B. Shi, Q. Lin, H. Yao and Y. M. Zhang, *Supramolecular Chemistry*, 2016, **28**, 720-726.
90. Y. Byun, J. Park and J. Kim, *International Journal of Environmental Analytical Chemistry*, 2016, **96**, 845-861.
91. Q. Zhang, J. Zhang, H. Zuo, C. Wang and Y. Shen, *Tetrahedron*, 2016, **72**, 1244-1248.
92. Y. Li, Q. Wang, X. Zhou, C.-y. Wen, J. Yu, X. Han, X. Li, Z.-f. Yan and J. Zeng, *Sensors and Actuators B: Chemical*, 2016, **228**, 366-372.
93. P. Sharma, D. S. Rana, A. Umar, R. Kumar, M. S. Chauhan and S. Chauhan, *Talanta*, 2016, **153**, 57-65.
94. A. Attar, L. Cubillana-Aguilera, I. Naranjo-Rodriguez, J. L. H.-H. de Cisneros, J. M. Palacios-Santander and A. Amine, *Bioelectrochemistry*, 2015, **101**, 84-91.
95. A. Paul, M. Perween, S. Saha, D. N. Srivastava and A. Das, *Physical Chemistry Chemical Physics*, 2015, **17**, 26790-26796.
96. R. Moosavi, A. Afkhami and T. Madrakian, *RSC Advances*, 2015, **5**, 15886-15891.
